# Impact of the pandemic on antimicrobial consumption patterns

**DOI:** 10.1017/ice.2020.1227

**Published:** 2020-09-23

**Authors:** Cristófer Farias da Silva, Caroline Deutschendorf, Fabiano Márcio Nagel, Camila Hubner Dalmora, Rodrigo Pires dos Santos, Thiago Costa Lisboa

**Affiliations:** 1 Infection Control Committee, Hospital de Clínicas de Porto Alegre, Porto Alegre, Brazil; 2 Programa de Pós Graduação em Ciências Pneumológicas/UFRGS, Porto Alegre, Brazil; 3 Department of Critical Care Medicine, Hospital de Clínicas de Porto Alegre, Porto Alegre, Brazil

*To the Editor—*Novel severe acute respiratory syndrome coronavirus 2 (SARS-CoV-2) impacts on economic, social, and healthcare systems. Uncertainties regarding coronavirus disease (COVID-19) promote concerns in choosing the best therapeutic strategy. Several drugs with antiviral effects were prescribed to treat COVID-19, but scientific evidence is not conclusive regarding benefit.

Unnecessary antimicrobial use may cause an increase in multidrug-resistant organisms.^1,2^ It is necessary to consider actions to prevent consequences that SARS-CoV-2 may have on antimicrobial use.^1,2^ Antibiotic stewardship is a strategy to promote the optimal use of antibiotics. SARS-CoV-2 probably changes the antibiotic consumption profile, and it is necessary to measure this difference.

Thus, our goal was to evaluate the impact of the pandemic on antimicrobial usage patterns comparing cohorts of SARS-CoV-2–positive and SARS-CoV-2–negative patients admitted in specific hospital locations.

## Methods

### Setting

Hospital de Clínicas de Porto Alegre, a 845-bed university, tertiary-care, public hospital is located in the city of Porto Alegre, southern Brazil. On March 20, 2020, Brazil declared recognition of community-based coronavirus transmission across the country. It is the local reference for hospitalization of patients with suspected or confirmed COVID-19. At the pandemic moment, areas for COVID-19 isolation were created in the intensive care unit (ICU), the emergency department, and clinical wards.

### Study design

A cross-sectional study was performed and data on antimicrobial consumption of May 2020 was included in our analysis. We adopted a “days of therapy” (DOT) methodology to measure antimicrobial consumption.^3^

### Data collection

All hospital antimicrobial data from administrative databases were included, except antibiotics that are not audited by the infection control committee. We conducted an overall analysis and cluster analysis in COVID-19 and non–COVID-19 ICU, emergency department, and clinical ward. We selected the most used antimicrobial drugs in each cluster. Units were coupled per similarity to compare COVID-19 and non–COVID-19 antibiotics consumption.

### Statistical analysis

We calculated antibiotics consumption based on DOT and adjusted per patient days (PD). We then compared this person–time rate with point estimates and confidence intervals for the incidence rate ratio considering Poisson distribution. The analysis was performed using Stata version 15.1 software (StataCorp, College Station, TX).

## Results

During the study period, we identified 18,079 PD. Of those, 9,065 were at clusters enrolled in the study, distributed in 1,028 PD at COVID-19 clusters (ICU 478, emergency department, 144, and clinical ward 406) and 8,037 PD non–COVID-19 clusters (ICU 1,165, emergency department, 1,407, and clinical ward 5,465).

The overall antibiotic use in the hospital during the study period was 73.0 DOT per 100 PD. The highest rate of antimicrobial use occurred in the COVID-19 emergency department (218.1 DOT per 100 PD), followed by the COVID-19 clinical ward (172.4 DOT per 100 PD), the COVID-19 ICU (134.3 DOT per 100 PD), the non–COVID-19 ICU (109.2 DOT per 100 PD), the non–COVID-19 emergency department (70.4 DOT per 100 PD), and the non–COVID-19 clinical ward (62.4 DOT per 100 PD) (Table 1). Comparing specific data between the COVID-19 emergency department and the non–COVID-19 emergency department, the incidence rate difference was 147.6 (95% CI, 123.1–172.1; *P* < .001); between the COVID-19 clinical ward and the non–COVID-19 clinical ward, the incidence rate difference was 110.0 (95% CI, 97.0–122.9; *P* < .001), and between the COVID-19 ICU and the non–COVID-19 ICU, the incidence rate difference was 25.1 (95% CI, 13.1–37.1; *P* < .001). These findings reveal a significantly higher rate of antimicrobial use in the COVID-19 units.

**Table 1.** Antimicrobial Consumption Measured by Days of Therapy (DOT) per 100 Patient Days (PD)

Antibiotic	Intensive Care Units, DOT per 100 PD			
	COVID-19	Non–COVID-19	COVID-19/Non–COVID-19, %	*P* Value
**All antibiotics**	134.4	109.2	123.1	<.001
Amoxicillin/clavulanate	22.4	5.0	448.0	
Azithromycin	21.3	4.8	443.8	
Cefepime	22.8	12.5	182.4	
Meropenem	11.7	23.8	49.2	
Piperacillin/tazobactam	3.3	9.1	36.3	
Vancomycin	5.6	18.8	29.8	
**Emergency department**				
All antibiotics	218.1	70.4	309.8	<.001
Amoxicillin/clavulanate	59.7	17.5	341.1	
Azithromycin	71.5	2.8	2553.6	
Cefepime	28.5	11.4	250.0	
Ceftazidime	3.5	2.9	120.7	
Cefuroxime	16.0	10.1	158.4	
Metronidazole	3.5	3.6	97.2	
Piperacillin/tazobactam	5.6	3.4	164.7	
**Clinical ward**				
All antibiotics	172.4	62.4	276.3	<.001
Amoxicillin/clavulanate	41.1	4.8	856.3	
Azithromycin	43.8	2.1	2,085.7	
Cefepime	17.0	7.9	215.2	
Cefuroxime	5.4	2.5	216.0	
Meropenem	6.2	8.6	72.1	
Piperacillin/tazobactam	7.9	6.6	119.7	
Sulfamethoxazole and trimethoprim	3.0	4.0	75.0	
Vancomycin	3.7	3.6	102.8	

Also, β-lactams and macrolides were used at a higher rate in COVID-19 clusters. Meropenem, a broad-spectrum antibiotic, was predominantly used in the non–COVID-19 units, mainly in the ICU, and was used less in non–COVID-19 clusters compared to other low-spectrum β-lactams such as amoxicillin/clavulanate and cefepime. Azithromycin exhibited the biggest relative differences between COVID-19 and non–COVID-19 clusters. We found a 2500% higher rate of azithromycin use in the COVID-19 emergency department and a 2000% higher rate of azithromycin use in the clinical ward. Beyond azithromycin, amoxicillin/clavulanate was used at a significantly higher rate in all COVID-19 clusters.

## Discussion

Our results show a difference in antimicrobial use in COVID-19 and non–COVID-19 areas. Overall antimicrobial consumption was more similar to that in the non–COVID-19 area. The COVID-19 cluster showed antibiotic use that was 2–3-fold higher than overall consumption. Nori et al^4^ found widespread antibiotic use in most hospitalized COVID-19 patients, similar to our study. Difficulty in differentiating COVID-19 from other infections may explain empirical treatment, but it does not justify maintenance on antibiotics after SARS-CoV-2 identification. In a recent review, Rawson et al^5^ did not identify data to support the COVID-19 association with bacterial/fungal coinfection.^5^ Furthermore, no studies have shown the benefit of antibiotics in COVID-19. Rather, many studies report drug resistance via inappropriate antimicrobial use and collateral effects that may cause harm to patients.

Our hospital has a stewardship program to control antibiotic use, but we do not have a protocol for COVID-19. We found some abuse of azithromycin and amoxicillin/clavulanate in the COVID-19 clusters. The excessive use of these drugs occurred mainly in COVID-19 emergency department and clinical ward. High antibiotic use in the COVID-19 area is likely related to low-quality scientific evidence, and social pressure may induce the wrong use of drugs related to anxiety related to curing COVID-19.

Abelenda-Alonso et al^6^ showed an increase in amoxicillin/clavulanate and broad-spectrum antibiotic use within the pandemic period between 2019 and 2020. These data are similar to antimicrobial consumption data in our study, which suggests the same global behavior regarding their prescription.

This study has some limitations. The DOT data were not individual data, which limited our analysis of clinical outcomes. Our period of analysis was short, and these findings may change over time. A longer study period is necessary to obtain more consistent data about antimicrobial use. However, the high rates of antimicrobial consumption in COVID-19 units is notable. Further work is needed to understand antimicrobial prescription behavior related to COVID-19.
